# Improved volumetric imaging in tomosynthesis using combined multiaxial sweeps

**DOI:** 10.1120/jacmp.v11i4.3331

**Published:** 2010-09-03

**Authors:** Jacob A. Gersh, David B. Wiant, Ryan C.M. Best, Marcus C. Bennett, Michael T. Munley, June D. King, Mahta M. McKee, Alan H. Baydush

**Affiliations:** ^1^ Department of Radiation Oncology Wake Forest University School of Medicine Winston‐Salem NC 27157 USA

**Keywords:** tomosynthesis, brachytherapy, cone beam CT

## Abstract

This study explores the volumetric reconstruction fidelity attainable using tomosynthesis with a kV imaging system which has a unique ability to rotate isocentrically and with multiple degrees of mechanical freedom. More specifically, we seek to investigate volumetric reconstructions by combining multiple limited‐angle rotational image acquisition sweeps. By comparing these reconstructed images with those of a CBCT reconstruction, we can gauge the volumetric fidelity of the reconstructions. In surgical situations, the described tomosynthesis‐based system could provide high‐quality volumetric imaging without requiring patient motion, even with rotational limitations present. Projections were acquired using the Digital Integrated Brachytherapy Unit, or IBU‐D. A phantom was used which contained several spherical objects of varying contrast. Using image projections acquired during isocentric sweeps around the phantom, reconstructions were performed by filtered backprojection. For each image acquisition sweep configuration, a contrasting sphere is analyzed using two metrics and compared to a gold standard CBCT reconstruction. Since the intersection of a reconstructed sphere and an imaging plane is ideally a circle with an eccentricity of zero, the first metric presented compares the effective eccentricity of intersections of reconstructed volumes and imaging planes. As another metric of volumetric reconstruction fidelity, the volume of one of the contrasting spheres was determined using manual contouring. By comparing these manually delineated volumes with a CBCT reconstruction, we can gauge the volumetric fidelity of reconstructions. The configuration which yielded the highest overall volumetric reconstruction fidelity, as determined by effective eccentricities and volumetric contouring, consisted of two orthogonally‐offset 60° L‐arm sweeps and a single C‐arm sweep which shared a pivot point with one the L‐arm sweeps. When compared to a similar configuration that lacked the C‐arm component, it is shown that the C‐arm improves the delineation of volumes along the transverse axis. The results described herein suggest that volumetric reconstruction using multiple, unconstrained orthogonal sweeps can provide an improvement compared with traditional cone beam CT using standard axial rotations.

PACS number: 87.57.nf

## I. INTRODUCTION

The integration of three‐dimensional imaging into the treatment planning of brachytherapy procedures reinforces the necessity for the actual treatment position to match the planning position. Cone beam computed tomography (CBCT) bases reconstruction on 2D projection images acquired while an X‐ray source and an image intensifier (or flat panel detector) rotates about a subject.^(^ ^1^ ^)^ This imaging modality allows for volumetric imaging while the patient remains in the treatment position. In surgical situations, the ability for a cone beam imaging system to be rotated 360° around a patient can be limited by gantry collisions with patient restraint systems, anesthesia equipment, IV tubes, catheters, the surgical table, and even the patient himself. These limitations give rise to the use of limited‐arc angle tomographic techniques. Tomosynthesis is one such technique where limited‐arc image sets of angular projection data are used to compile three‐dimensional tomographic reconstructions.^(^ ^2^ ^)^ This study explores the volumetric reconstruction fidelity attainable using tomosynthesis with a clinically‐available digital kV imaging system which can rotate isocentrically and with multiple degrees of mechanical freedom. More specifically, we seek to optimize volumetric reconstruction fidelity by combining multiple limited‐angle orthogonally‐offset rotational image acquisition sweeps. It is expected that without the limitation to remain in an axial‐only rotation (the classical motion of a CBCT imager), this system can allow for a more complete filling of frequency space along the transverse axis and therefore provide improved volumetric imaging over limited arcs. This study determines suitable radiographic image‐sweep combinations that can be performed in a clinical setting for three‐dimensional image reconstruction using tomosynthesis.

There are several aspects of tomosynthesis that favor its medical application: its ability to acquire volumetric data rapidly, provide a very high in‐plane resolution, and impart a lower‐than‐CT dose to the patient.^(^ ^3^ ^,^ ^4^ ^)^ There are, however, several disadvantages inherent to this technique. A primary limitation in tomosynthesis is that, since limited arc acquisition results in incomplete sampling in frequency space, imaged structures that lie distal with respect to the axis‐of‐rotation can be superimposed with those which fall more medial, resulting in the reduction of volumetric reconstruction fidelity.^(^ ^5^ ^,^ ^6^ ^)^ This effect is shown to increase as the distance from the rotational isocenter is increased.^(^ ^3^ ^)^ Volumetric reconstruction fidelity is also reduced as the angular image sampling density (projections per angle) is reduced.^(^ ^7^ ^,^ ^8^ ^)^ This can be seen with the appearance of streak artifacts in reconstructions based on low image sampling density projections in CBCT and tomosynthesis modalities.

Two main factors of the image acquisition geometry that affect the fidelity of volumetric reconstruction include acquisition angle and angular projection density. As the acquisition angle (the total planar angle subtended during image acquisition) increases, the filling of the frequency space helps to complete the high‐frequency component of objects in the Fourier space, and edge delineation in volumetric reconstruction improves. As the density of projections increase, the number of degrees‐per‐image decrease and a more complete filling of frequency space occurs; however, the dose to the patient increases. It should also be noted that, if the angular separation is decreased low enough, projections provide an increased amount of redundant low‐frequency information (which can result in image degradation). Several studies have investigated these geometric factors with respect to the role they play in reducing volumetric reconstruction fidelity.^(^ ^8^ ^,^ ^9^ ^)^ Sechopoulos et al.^(^ ^10^ ^)^ concluded that optimal geometry for breast tomosynthesis requires a 60° acquisition angle and an angular projection density of 4.6° per projection. Similarly, Soimu et al.^(^ ^3^ ^)^ suggested acquisition steps not be greater than 6°, concluding that 4° per projection was adequate in providing a compromise between resolution and imaging dose. With the results of these studies in mind, the multiple‐sweep reconstructions analyzed in this study are based on 60° rotation sets acquired with angular projection densities between 1.66° per projection and 5° per projection.

The purpose of this study is to determine combinations of projection acquisition sweeps that allow for the highest volumetric reconstruction fidelity in tomosynthesis‐based volumetric reconstructions. Following the volumetric reconstruction of a geometrically diverse set of image acquisition sweeps, an off‐axis contrasting sphere is analyzed using multiple metrics and compared to a gold standard CBCT reconstruction. Since the intersection of a reconstructed sphere and an imaging plane is ideally a circle with an eccentricity of zero, the first metric presented compares the effective eccentricity of intersections of reconstructed volumes and imaging planes. As another metric of volumetric reconstruction fidelity, the volume of one of the contrasting spheres was determined using manual contouring. By using these metrics and comparing to a CBCT reconstruction, we can gauge the volumetric fidelity of reconstructions, which are based on different sets of geometric combinations.

## II. MATERIALS AND METHODS

**Figure 1:**
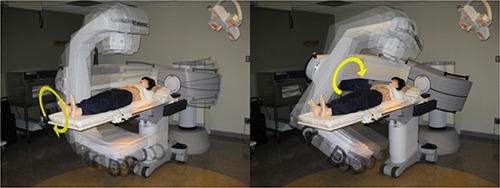
Demonstration of the unique motion of the Digital Integrated Brachytherapy Unit, or IBU‐D while rotating around the PIXY anthropomorphic phantom: (left) the motion of the L‐arm, which rotates the kV tube‐and‐detector axially around the patient; (right) the motion of the C‐arm, which rotates the kV tube‐and‐detector orthogonally with respect to the L‐arm, along the inferosuperior axis of the patient.

Images were acquired using the Digital Integrated Brachytherapy Unit, or IBU‐D (Nucletron, B.V., Veenendaal, The Netherlands). Shown in Fig. 1, this device is designed specifically to provide conventional film, digital and fluoroscopic radiography for treatment planning, patient positioning, and applicator and source positioning in brachytherapy procedures. This imaging system consists of a kV X‐ray tube attached to a 41 cm by 41 cm XD‐1640AN amorphous silicon (a‐Si) digital flat‐panel detector (PerkinElmer Optielectronics, Fremont, CA). This system provides digital radiographic images (or projections) in 16‐bit grayscale depth, with a size of 1024×1024 pixels, at a resolution of 0.4 mm/pixel. Unique to this clinically available imaging system is its capabilities of rotating around multiple axes while maintaining a common isocenter. The C‐arm is named for the shape it makes, with one end of the “C” containing the kV X‐ray tube and the other end containing the flat‐panel digital detector. The L‐arm is named for the shape it makes with the C‐arm attached at one end and the base of the unit at the other end. The L‐arm rotates around the axial plane of the patient (or L‐axis). The C‐arm rotates about the C‐axis, which orthogonally intersects the L‐axis. The L‐arm and C‐arm are positioned manually with the aid of digital angular‐location readouts. As part of a research agreement with Nucletron, the developers of the IBU‐D created an experimental version of the image acquisition software, which allows for cine capture of digital fluoroscopic images at two frames per second. The X‐ray tube is 80 cm from the isocenter of the system. The flat‐panel imager can move closer to and farther from the isocenter (and X‐ray tube). However, in order to minimize the likelihood of table collisions and maximize the arc length of the rotating system, the imager is set to rotate 40 cm from the isocenter for this study. With a source‐to‐detector distance (SDD) of 120 cm and maintaining fully open X and Y collimating shutters, the cone beam angle is approximately 10°.

The phantom used in this study is a water‐filled Jaszczak SPECT Phantom (Biodex Medical Systems, New York, NY) containing three sphere‐and‐wand structures: a hollow air‐filled sphere, a 16.$8\;{\text{cm}}^{3}$ contrast‐filled sphere, and a solid acrylic sphere, arranged medial‐to‐distal, respectively. This cylindrical phantom has an interior diameter of 21.6 cm and an interior height of 18.6 cm. The contrast used in this study was a 15% solution of Conray 30 (Mallinckrodt Pharmaceuticals., St. Louis, MO) and saline. When imaged with CT, this particular sphere had an average CT number of 650. The table top is constructed of wood in order to reduce the contrast at table‐to‐air interfaces, subsequently reducing the streak artifacts that appear in low angular projection density reconstructions such as those described herein.^(^ ^11^ ^)^

Reconstructions are performed using filtered backprojection (FBP) using the Feldkamp‐Davis‐Kress algorithm, a method of image reconstruction commonly used in tomographic imaging modalities such as CT.^(^ ^12^ ^–^ ^13^ ^,^ ^14^ ^)^ The mathematics defining the use of FBP for CBCT are described in depth by Feldkamp et al.^(^ ^14^ ^)^ and summarized well by Sakamoto et al.^(^ ^15^ ^)^ In the current implementation of this technique, projection data are provided by the onboard flat‐panel detector as the imaging system rotates isocentrically around the subject. Each projection is stored as an image file containing image acquisition data, including the L‐arm location, the C‐arm location, and the distance between the kV X‐ray tube and the flat‐panel detector. The reconstructed volumes are normalized and output in DICOM format. Postprocessing of these normalized images is limited to temporary adjustments of window and level, an adjustment left to the discretion of the observers at the time of volumetric contour analysis. The reconstruction code was designed using MATLAB Version 7.7.0 (The MathWorks, Natick, Massachusetts).

**Figure 2:**
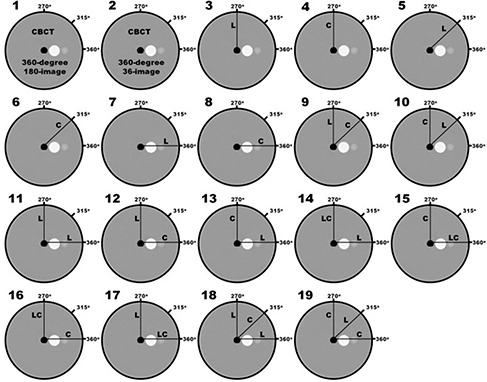
Diagrams of the sweep configurations analyzed in the current study. Line segments labeled as “L” represent the center‐of‐rotation for L‐sweeps (which occur in the plane of the illustrated circle). Line segments labeled as “C” represent the center‐of‐rotation for C‐sweeps (which occur perpendicular to plane of the illustrated circle). The CBCT‐based reconstruction shown in Configuration 1 serves as the gold standard for comparing volumetric reconstruction fidelity in the current study.

Initially, 19 combinations of image projections were analyzed (all shown in Fig. 2). This set contained two full‐sweep CBCT sets, six single limited‐arc sets, five double limited‐arc sets, and six triple limited‐arc combinations. These combinations included variations in angular separation between sweeps, as well as variations in sweep direction (axial or transaxial). Configuration 1 is a combination of 180 images, acquired during a 360° L‐arm sweep with an average image separation of 2° per image. This CBCT reconstruction represents the upper limit of three‐dimensional fidelity capable when using this imaging system. This CBCT reconstruction serves as the gold standard for this study and to which subsequent reconstructions are compared. This CBCT image is also used because it is the exact same resolution as the tomosynthesis images, as opposed to the CT image that was acquired using a different resolution. The other sweep configurations analyzed in this study each consisted of 36 images. Configuration 2 is similar to Configuration 1 in that it is comprised of a 360° sweep of images; however, this Configuration is made up of only 36 images (approximately a 10° image separation).

Configuration 3 is a 36‐image 60° L‐arm sweep, centered at the point L=270° (where the X‐ray tube is at the highest point in its axial rotation). During this rotation, the C‐arm is kept orthogonal to the L‐arm axis. Configuration 4 is a 36‐image 60° C‐arm sweep, centered at the point C=270° (where the X‐ray tube is at the highest point in its axial rotation). During this rotation, the L‐arm is kept orthogonal to the C‐arm axis. Configurations 3 and 4 are orthogonal to each other. Configurations 5, 6, 7 and 8 are all single 60° sweeps as depicted in Fig. 2. Configurations 9–13 are combinations of two 60° sweeps each consisting of 18 images (maintaining a total image set of 36). Configurations 14–19 are combinations of three 60° sweeps each consisting of 12 images (again maintaining a total image set of 36). Limited‐arc acquisition sweeps were performed with an angular subtension of 60°, since this was the greatest symmetric rotation allowed during a C‐arm sweep (due to table collision). Images were acquired with tube and current settings of 120 kVp and 3.25 mAs, respectively.

**Figure 3:**
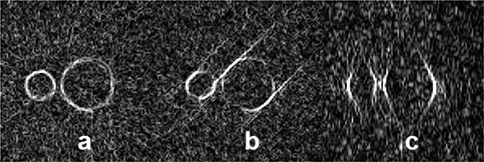
Canny‐Deriche edge detected axial sliced: Configurations 1 (a) and 18 (b) are examples where geometrically‐closed structures were observed; Configuration 3 (c) was considered geometrically opened. Incomplete delineations (such as is the case in (c)) are not possible, since a vertical profile through the center of the sphere would show no deviation apart from noise.

For all 19 reconstructions and a CT reconstruction, a Canny‐Deriche^(^ ^16^ ^–^ ^19^ ^)^ edge detection filter was applied to the axial slice that lies equatorially in the contrasting sphere. As an example, Fig. 3 shows several zoomed‐in images of these axial slices. Figures 3(a) and 3(b) (Configurations 1 and 18, respectively) are examples of geometrically closed structures, while image 3(c) (Configuration 3) is considered geometrically open. Automated routines based on incomplete delineations (such as is the case in image 3(c)) are not possible, since a vertical profile through the center of the sphere would show no deviation apart from noise.

**Figure 4:**
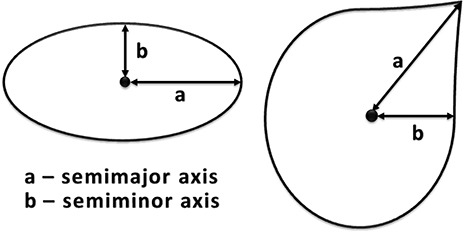
Illustration of the geometric parameters used to calculated the effective eccentricity (as described by equation where the semimajor axis is defined as half the length of longest chord intersecting the geometric center and the semiminor axis is defined as half the length of shortest chord intersecting the geometric center). The ellipse on the left shows how eccentricity can be classically calculated, where the semimajor and semiminor axes are orthogonal. The irregular shape on the right shows how the ‘effective eccentriciy’ can be calculated, where the semimajor and semiminor axes do not require orthogonality.

With the contrasting sphere in the remaining reconstructed volumes being ellipsoidal, the eccentricity in central intersecting slices is used to quantify the deviation from a true spherical object, which will have zero eccentricity (circular) areas of intersection with intersecting planes. The use of eccentricity in its purely mathematical form would be, however, incorrect since the outlines determined using Canny‐Deriche are irregular in shape and cannot be made mathematically to fit without the use of smoothing and regression algorithms. Instead, an ‘effective eccentricity’ is used to quantify the closeness of a shape to a circle. As shown in Eq.(1) and illustrated in Fig. 4, the definition is identical to eccentricity. However, instead of ‘a’ and ‘b’ being the semimajor and semiminor axes, respectively and orthogonally, ‘a’ and ‘b’ are defined as half the length of longest chord intersecting the geometric center and half the length of shortest chord intersecting the geometric center, respectively. These are not necessarily orthogonal. This metric is useful since it requires no smoothing of delineation data, it is simultaneously implementable across many shape constructs, and it can easily reveal subtle differences in shapes such as, for example, a sphere and a sphere containing a small dimple. In this study, chords extend from the center of the outline, which has an average thickness of 2 pixels. Table 1 shows the results of eccentricity measurements in the axial plane. Since the CT voxels were not cubes (elongated in the z‐direction), the effective eccentricity was only calculated in the axial plane, where pixels were squares:

**Table 1:** Effective eccentricities (Eq. (1)) calculated from axial, coronal and sagittal slices through the center of the contrasting sphere. These were calculated for only the configurations where a Canny‐Deriche edge detection algorithm could provide a closed geometric structure. Effective eccentricities in the axial plane were calculated for all these configurations. Those with effective eccentricities of less than 0.5 were also analyzed in the coronal and sagittal planes.

*Configuration*	$\varepsilon_{{eff}^{\prime }}$ *Axial*	$\varepsilon_{{eff}^{\prime }}$ *Coronal*	$\varepsilon_{{eff}^{\prime }}$ *Sagittal*
CT	0.03838	x	x
1	0.05573	0.08486	0.09882
2	0.07377	0.08648	0.1055
9	0.7031	x	x
10	0.7826	x	x
11	0.3447	0.2179	0.1781
12	0.4701	0.2799	0.2004
13	0.1927	0.2171	0.1517
14	0.1046	0.1837	0.1347
15	0.6132	x	x
16	0.6568	x	x
17	0.3156	0.3366	0.5137
18	0.3083	0.3335	0.2133
19	0.6493	x	x

(1) $$\begin{array}{rl} & \varepsilon_{eff}=\frac{\sqrt{a^{2}-b^{2}}}{a}\\ & a=half\;the\;length\;of\;longest\;chord\;intersecting\;the\;geometric\;center\\ & b=half\;the\;length\;of\;shortest\;chord\;intersecting\;the\;geometric\;center \end{array}$$

An effective eccentricity (from now on referred to as simply eccentricity) of 0.5 was chosen to be a value above which further analysis would not proceed. For configurations with axial eccentricities below 0.5, the Canny‐Deriche filter was applied to equatorial coronal slices as well as equatorial sagittal slices.

**Figure 5:**
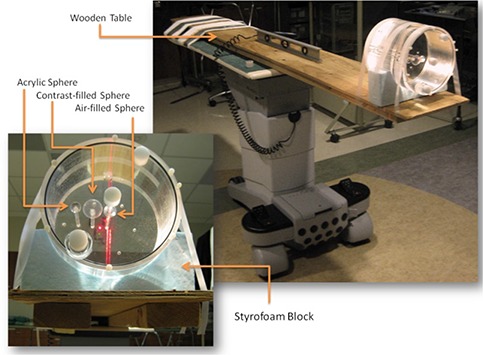
The phantom and tabletop used in the current study.

The remaining eight configurations (and the CT reconstruction) were also analyzed using volumetric contouring as a metric for the comparison of volumetric reconstruction fidelity. Contouring is used regularly in radiation therapy treatment planning, where regions‐of‐interest are manually delineated within a stack of reconstructed images. After determining the number of pixels bound by the delineated region, the volume can be calculated by resolution‐dependent conversion. In the current study, the volume of the contrast‐filled middle sphere (as shown in Fig. 5) was contoured and calculated for each reconstruction. Contouring was performed using two programs: MIPAV (Medical Image Processing, Analysis, and Visualization) Version 4.3.1 and Eclipse External Beam Planning System Version 7.3.10 (Varian Medical Systems, Inc., Palo Alto, CA). Since the volume determined from manual contouring is inherently subjective, multiple observers were used and multiple measurements were performed using both the experimental contouring program (MIPAV) and the clinical contouring program (Eclipse). This study included the use of five observers, each performing between 9 and 15 volumetric contours for each of the eight configurations (as well as for the CT reconstruction). Though automatic contouring algorithms are available in each of these programs, every contour was performed manually in order to maintain the inherent subjectivity of the metric.^(^ ^20^ ^)^ Though radiation oncology professionals routinely contour in the axial plane, there is the possibility that an alternate traversal might give rise to a better region‐of‐interest delineation, especially for techniques that are not fully sampled, such as tomosynthesis. Therefore, each volume was contoured during slice‐by‐slice traversals along each of the three imaging planes: axial, coronal and sagittal. Prior to analysis, volumetric calculations were performed in a single observer study between the two programs. No difference between the two programs was observed. Since MIPAV's calculations were based on a minimum voxel size of approximately 0.05cm×0.05cm×0.05cm and Eclipse calculations were based on a minimum voxel size of 0.1cm×0.1cm×0.1cm, the precision of volumes used in the study does not exceed 0.$1\;{\text{cm}}^{3}$.

## III. RESULTS

A 512×512×512 voxel reconstruction based on 36 images (with 16‐bit grayscale depth and 1024×1024 projection matrix) required approximately 35 minutes on a 64‐bit Windows workstation running a 3.0 GHz Intel Xeon quad‐core processor with 8 GB of RAM. As of publication, this code was not optimized for speed, a necessary step prior to clinical implementation. The resolution of the reconstructions performed using the code developed during this study is 0.5 mm/pixel.

**Figure 6:**
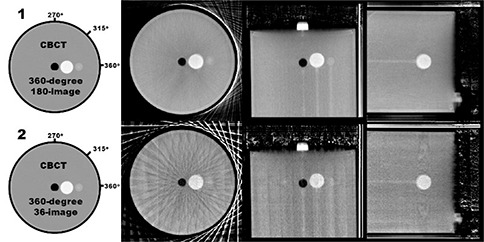
Contrasting sphere slices in the axial, coronal and sagittal planes (from left to right, respectively) of reconstructions based on the CBCT sweep configurations.

**Figure 7:**
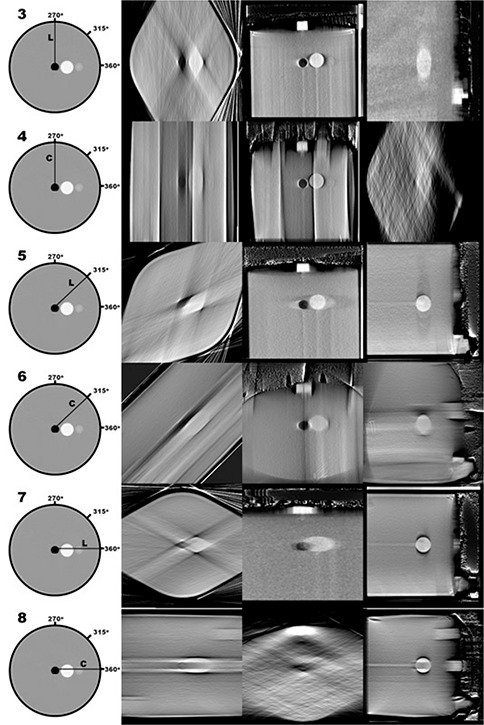
Contrasting sphere slices in the axial, coronal and sagittal planes (from left to right, respectively) of reconstructions based on the single‐sweep configurations.

**Figure 8:**
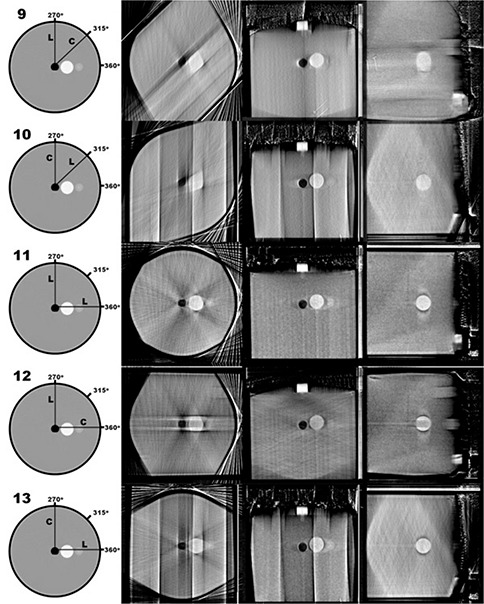
Contrasting sphere slices in the axial, coronal and sagittal planes (from left to right, respectively) of reconstructions based on the double‐sweep configurations.

**Figure 9:**
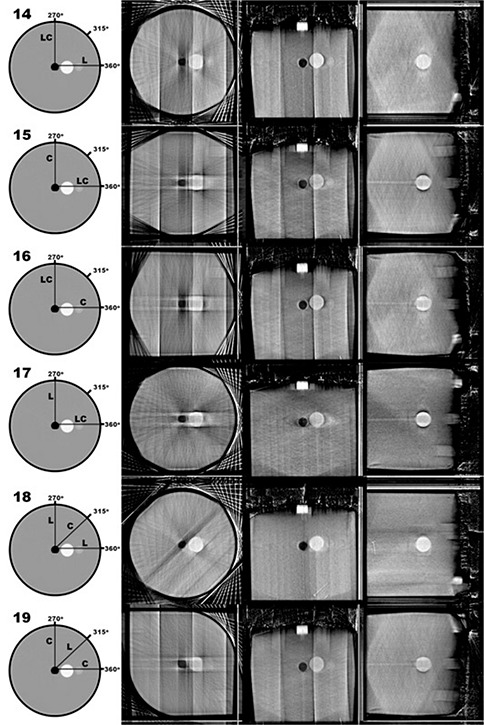
Contrasting sphere slices in the axial, coronal and sagittal planes (from left to right, respectively) of reconstructions based on the triple‐sweep configurations.

Figures 6–9 show central slices of the 19 reconstructions analyzed in the current study. Each row of images show equatorial slices of the contrast sphere in the axial, coronal and sagittal planes (from left to right, respectively). For single‐sweep acquisitions (Configurations 3–8, shown in Fig. 7) edge detection does not result in a closed geometric structure as a result of the incompleteness of data in frequency space. Therefore, further analysis was not performed using these data. Table 1 shows the calculated eccentricities of the remaining 13 reconstructed configurations and the CT image. A large eccentricity (greater than 0.5) was calculated in the central axial slice for Configurations 9, 10, 15, 16 and 19; therefore, further analysis using these data did not proceed. For the remaining nine reconstructed configurations, the eccentricity was calculated in coronal and sagittal planes, and shown in Table 1. Of the tomosynthesis sweeps, Configuration 14 had the lowest eccentricity in all imaging planes.

Table 2 shows the results of volumetric contour analysis of the nine reconstructed configurations whose eccentricity in the axial plane was less than 0.5. This Table also shows the contoured volumes for the CT reconstruction. In order to show an example of a low‐volumetric fidelity reconstruction, the contoured volume of the single‐arc 60° L‐arm sweep (Configuration 9) is also shown in this Table. No statistically significant difference was found upon changing the contoured imaging plane; therefore, the data in the table represent the average of all data acquired through volumetric contouring along each image plane. The contoured volumes in Configurations 11 and 14 both deviated by less than 1% in comparisons with the gold standard, CT reconstruction, and the actual volume of the contrasting sphere.

**Table 2:** Average volumes of the contrast‐filled sphere, as determined from volumetric contouring. Following multiple repeat measurements to account for the inter‐ and intra‐observer, each volume is compared to the measured volume in the gold standard (Configuration 1).

*Configuration*	*Average Volume (cm^3^)*	*Std Dev (cm^3^)*	*Pct Std Dev (%)*	*Pct Dev from Gold (%)*	*Pct Dev from CT (%)*	*Pct Dev from Actual (%)*
CT	16.81	0.1307	0.7775	0.9127	x	0.05952
Actual	16.80	NA	NA	1.012	0.05952	x
1 (Gold)	16.97	0.3772	2.223	x	0.9127	1.012
2	16.33	0.5361	3.283	3.759	2.881	2.798
9	19.89	3.406	17.12	17.21	18.27	18.39
11	16.92	0.4633	2.738	0.2751	0.6652	0.7143
12	17.52	0.6572	3.751	3.246	4.189	4.286
14	16.89	1.125	6.661	0.4890	0.4192	0.5357
16	17.99	0.6714	3.732	6.020	6.988	7.083
18	16.65	0.7762	4.662	1.897	1.0018	0.8929
19	17.70	0.8356	4.721	4.299	5.251	5.357

Table 3 shows the contrast ratios measured on central axial sliced for the 19 configurations. Contrast ratio is the ratio of the difference in average pixel from a central circular region‐of‐interest of the highest pixel intensity (contrasting sphere) and average pixel from a central circular region‐of‐interest of the lowest pixel intensity (air‐filled sphere) to the average pixel from a central circular region‐of‐interest of the highest pixel intensity (contrasting sphere).

**Table 3:** Contrast ratios measured on central axial sliced for the 19 configurations. Contrast ratio is the ratio of the difference in average pixel from a central circular region‐of‐interest of the highest pixel intensity (contrasting sphere) and average pixel from a central circular region‐of‐interest of the lowest pixel intensity (air‐filled sphere) to the average pixel from a central circular region‐of‐interest of the highest pixel intensity (contrasting sphere).

*Configuration*	*Contrast Ratio*
1	0.951
2	0.892
3	0.899
4	0.797
5	0.757
6	0.846
7	0.470
8	0.210
9	0.916
10	0.895
11	0.860
12	0.840
13	0.875
14	0.903
15	0.772
16	0.877
17	0.844
18	0.926
19	0.789

## IV. DISCUSSION

The effect of limited arc angle is visually apparent in Fig. 7. In single sweep reconstructions, the center‐most slice that lies perpendicular to the center of rotation has the highest volumetric reconstruction fidelity. As the distance from the isocenter increases, the system cannot provide enough spatial information concerning edge detection of central structures, and these objects appeared smeared in successive image slices. In Configuration 3, the image acquisition sweep is centered perpendicular to the coronal plane, hence the higher‐quality central slice. When this reconstruction is viewed axially and sagittally, we can see the effects of this smearing. Streak artifacts are not as prominent, since this effect is reduced by increased angular sampling density. As shown in Table 2, this configuration resulted in the highest inaccuracy in volumetric measurement. For single‐sweep acquisitions (Configurations 3–8, shown in Fig. 7), edge detection does not result in a closed geometric structure as a result of the incompleteness of data in frequency space.

Comparing the effective eccentricity of the intersection of the contrasting sphere and the central axial plane, CT provided the lowest value, followed by Configuration 1 (180‐image CBCT), and then Configuration 2 (36‐projection CBCT). These results parallel the expected results: CT would provide the highest volumetric reconstruction fidelity, followed by a higher angular image density CBCT, followed by a lower angular image density CBCT. These results also help substantiate the use of effective eccentricity as a comparative metric. It is worth noting that a visual comparison between Configuration 1 and 2 (as shown in Fig. 6) shows the effect that a reduction in sampling density (undersampling) has on image quality. The large angular separation between successive projections causes resultant discontinuities in the frequency domain, which appear in the spatial domain as high‐contrast streak artifacts. Though the low image projection sampling causes streak artifacts, the resultant volumetric reconstruction fidelity of the image still remains acceptable. We conclude that the projection density is acceptable in both the two‐ and three‐sweep combinations of 60° acquisition sweeps.

Eccentricities greater than 0.5 – the value at which a configuration was deemed unfit – were observed where sweep axes were 45° apart (with the exception of Configuration 18). These data suggest that multiaxial sweeps need to be a greater arc distance from one another, where image degradation is caused by redundant sampling in frequency space. In a preliminary study, this was also shown when L‐arm/C‐arm sweeps that pivoted around a single point showed a large reduction in volumetric reconstruction fidelity. In Configuration 18, this effect is most likely offset by the inclusion of an orthogonal L‐arm sweep. The redundancy effect was also noticed in reconstructions consisting of multiple C‐arm sweeps. With this in mind, it is concluded that configurations contain only a single C‐arm sweep (in addition to L‐arm sweeps).

Configuration 14 was able to provide higher volumetric reconstruction fidelity along the transverse axis, as shown in the calculated eccentricities in the coronal and sagittal planes, while maintaining high volumetric reconstruction fidelity in the axial plane. This configuration also yielded contour volumes closest to the CT‐measured volume and the actual volume. With respect to eccentricity and volumetric comparison to CT and the actual volume (as given in the phantom's specifications), Configuration 14 is numerically superior, slightly more so than Configuration 11. These configurations differ in two ways. Since Configuration 11 is a two‐sweep set, its angular image density is higher than in the three‐sweep Configuration 14. Also, Configuration 11 consists of only L‐arm sweeps, while Configuration 14 includes a C‐arm sweep. It is concluded that the addition of the C‐arm improves the delineation of volumes when the contrasting sphere was located on the axis about which the C‐arm rotates.

Since the ultimate goal of this project involves clinical implementation, smaller solid angle subtension of the imaging gantry (in all rotational degrees of freedom) is desired in order to reduce possible collision with the patient and the clinical surroundings. Therefore, the angular separation between two offset sweeps was limited to 90°. Since this separation is in reference to the center‐most projection angle, the largest sweep studied subtends a 150° arc (which includes a 30° discontinuous arc in the middle). It is possible that larger separations could provide better volumetric reconstruction fidelity; however, we chose to maintain the clinical viability of this technique and limit this geometric factor. The methods described herein, though presented as relating to the IBU‐D, can be applied to any imaging modality which can isocentrically rotate, unconstrained to a single plane. An example of one such application would be gantry‐mounted digital on‐board imaging systems which provide CBCT capabilities for patient positioning purposes. This technique would take advantage of the rotation of the treatment couch to provide non‐coaxial projections for image reconstruction.

## V. CONCLUSIONS

This work represents the first study of the Digital Integrated Brachytherapy Unit used in the capacity of a CBCT imager with tomosynthesis capabilities. The results described herein suggest that volumetric reconstruction using multiple, unconstrained orthogonal sweeps can provide an improvement compared with traditional cone beam CT. The results of this study suggest image acquisition parameters for improving volumetric reconstruction and delineation for this particular geometric phantom and setup. The configuration which yielded the highest overall volumetric reconstruction fidelity, as determined by effective eccentricities and volumetric contouring, consisted of two orthogonally‐offset 60° L‐arm sweeps and a single C‐arm sweep which shared a pivot point with one the L‐arm sweeps. From these studies, we conclude that the addition of the C‐arm improves the delineation of volumes when the contrasting sphere was located on the axis about which the C‐arm rotates. In a clinical procedure, the orientation of the C‐arm's rotational axis would be chosen based on the location of major contrasting objects. For example, in a procedure where the bladder or rectum of a supine patient is filled with contrasting agent, a configuration with an anteriorly‐centered C‐arm sweep would likely yield superior imaging performance. For imaging a patient with a hip prosthesis, a laterally‐centered C‐arm sweep would likely yield superior imaging performance. Additionally, in surgical situations, the described tomosynthesis‐based system could provide high volumetric reconstruction fidelity without requiring patient motion, even with rotational limitations present. If used during brachytherapy procedures, the techniques described herein could provide better matching between patient treatment and planning positions, leading to more accurate treatments.

## ACKNOWLEDGEMENTS

Research supported in part by NIH T32‐CA113267 and Nucletron, B.V. The authors would like to thank Mandy M. Parker, Lorne Kerley, Yuchuan Wei, J. Daniel Bourland and Kevin A. Muhanji for their assistance and support during this project.
